# Anticancer Properties of Essential Oils and Other Natural Products

**DOI:** 10.1155/2018/3149362

**Published:** 2018-03-25

**Authors:** K. Blowman, M. Magalhães, M. F. L. Lemos, C. Cabral, I. M. Pires

**Affiliations:** ^1^School of Life Sciences, University of Hull, Hull HU6 7RX, UK; ^2^REQUIMTE/LAQV, Group of Pharmaceutical Technology, Faculty of Pharmacy, University of Coimbra, 3000-548 Coimbra, Portugal; ^3^Marine and Environmental Sciences Centre (MARE), Instituto Politécnico de Leiria, ESTM, 2520-630 Peniche, Portugal; ^4^Centre of 20th Century Interdisciplinary Studies (CEIS20) & Center for Neuroscience and Cell Biology and the Institute for Biomedical Imaging and Life Sciences (CNC.IBILI), Faculty of Pharmacy, University of Coimbra, 3000-548 Coimbra, Portugal

## Abstract

Essential oils are secondary metabolites with a key-role in plants protection, consisting primarily of terpenes with a volatile nature and a diverse array of chemical structures. Essential oils exhibit a wide range of bioactivities, especially antimicrobial activity, and have long been utilized for treating various human ailments and diseases. Cancer cell prevention and cytotoxicity are exhibited through a wide range of mechanisms of action, with more recent research focusing on synergistic and antagonistic activity between specific essential oils major and minor components. Essential oils have been shown to possess cancer cell targeting activity and are able to increase the efficacy of commonly used chemotherapy drugs including paclitaxel and docetaxel, having also shown proimmune functions when administered to the cancer patient. The present review represents a state-of-the-art review of the research behind the application of EOs as anticancer agents both* in vitro* and* in vivo*. Cancer cell target specificity and the use of EOs in combination with conventional chemotherapeutic strategies are also explored.

## 1. Introduction

Whilst some synthetic compounds unequivocally have an important role in disease prevention and therapy, there is also an extensive collection of naturally existing compounds that have been exploited for their unique medicinal purposes [1]. The use and demand of natural compounds have been increasing worldwide, showing their importance, which can be attributed to relevant medicinal properties [2]. Essential Oils (EOs) and other phytoproducts are examples of natural products that have gained interest, mainly due to their suitable chemical characteristics and biological activities [3].

As stationary organisms, plants have evolved a diverse range of protective mechanisms to lessen their vulnerability against external threats. These mechanisms can be classified as physical and chemical defenses. Physical deterrents include protective structural characteristics, which include waxy barriers, spikes, and “hair-like” trichomes, which release chemical compounds [4]. Chemical defense mechanisms include, for example, the production of a range of defensive metabolites bioactive compounds with the capability to repulse herbivores or even to target their endocrine and nervous system [5, 6]. These include EOs, enzymes, tannins, and flavonoids, amongst others. Importantly, these compounds are also of pharmacological interest.

EOs are complex and multifunctional substances with plant origin, which have been used for thousands of years for their role in the prevention and treatment of various ailments [3, 7, 8]. Chemically, EOs are aromatic plants secondary metabolites with several roles: defense against herbivores, insects, and microorganisms; communication with plants of the same species; and signaling within the plant in response to environmental stimuli [5]. As each plant species or subspecies has evolved to protect itself from a particular predator or group of predators, each plant produces its own specific “signature” mixture of EO chemical constituents [5, 7]. This can contain from 20 to 60 constituents at varying concentrations, with two or three primary constituents (20–70%) [9, 10].

### 1.1. Chemical Composition of EOs

There are approximately 3000 EOs, from over 2000 different plants, with around 300 EOs possessing known biomedical features [2, 10, 11]. Together with the plant species, the developmental stage (flowering, fruiting) and aromatic compound extraction methods have a direct influence on the composition of EOs, which explains the variability of components in the reported EOs [12].

**Figure 1:**
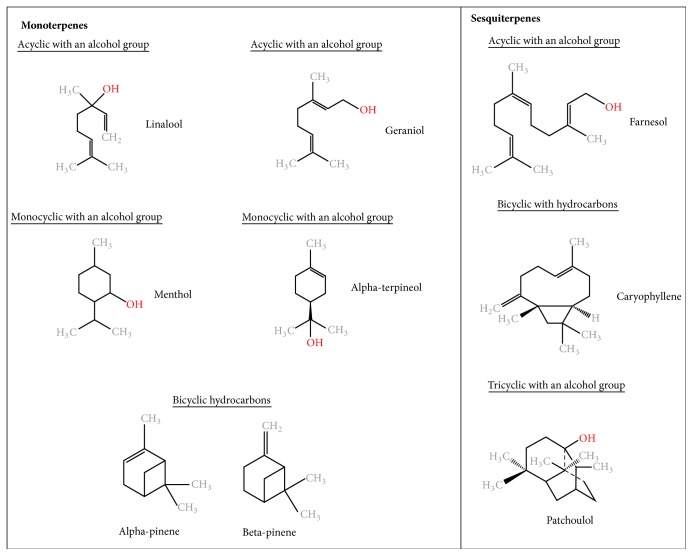
Chemical structures of essential oil constituents.

Based on their chemical compositions, EOs are broadly categorized into oxygenated compounds and hydrocarbons [9]. Oxygenated compounds include esters, aldehydes, ketones, alcohols, phenols, and oxides. Other active groups include aromatics and sulfur-containing components [9–12, 24]. Hydrocarbon compounds are composed of one specific chemical group called terpenes (Figure 1) [9]. These are composed of varying numbers of isoprene units (C_5_). Monoterpenes (C_10_) and sesquiterpenes (C_15_) are the main terpenes, although the isoprene chains may also include diterpenes (C_20_). Monoterpenes contribute to 90% of EO overall constituents [9]. Both monoterpenes and sesquiterpenes offer a large variety of structures through adjoining with other biologically active functional groups (monoterpenoids), and chemical rearrangement and addition of oxygenated groups (sesquiterpenoids) [9]. Terpenes may also be acyclic, monocyclic, or bicyclic and may contain an aromatic group [9]. The longer the isoprene chain, the more the chemical variations possible [9, 24]. The structures of several medicinally important terpenes are illustrated in Figure 1.

Due to the large range and complex blend of EOs constituents, as well as their many functional groups, it is thought that EOs do not possess a specific single cellular target, with each complex mixture initiating different cellular effects through their major constituents [9, 10]. However, it is important to consider the minor constituents of an EO, and the different cellular effects exhibited when the constituents are combined in the EO blend versus the isolated constituents. A study performed by Santana-Rios and coworkers (2001) isolated the main constituents of both white and green tea and created an artificial “mixed” tea with a total of 9 main constituents [25]. The artificial tea exhibited a lesser antimutagenic effect than the whole tea extracts in the* Salmonella* assay in the presence of *N*-hydroxy-IQ, a potent mutagen. Furthermore, it has been shown that EOs extracted from the tea tree, eucalyptus, and thyme plants reduced* Herpes simplex* virus- (HSV-) 1 viral infectivity by more than 96% in an* in vitro* study through inactivation of virus-free particles, with the combined EO constituents more effective than the isolated counterparts [26]. Recent studies also have been pointing out the therapeutic potential of the individual constituents of EOs, such as the work of Dias and colleagues (2017), which showed a possible association between the oxygenated monoterpenes of EOs extracted from* Lavandula luisieri* and* Cymbopogon citratus* and the antifungal activity against dermatophytes [27]. This was because an inhibitory effect was observed on the conidial germination, demonstrating the strong antifungal activity of these EOs components [27]. The mentioned studies indicate that minor constituents possess both synergistic and antagonistic activities on the major constituents, playing an important role in the overall properties of EOs on a variety of cell types.

### 1.2. EOs as Therapeutic Agents

Only 5 to 15% higher order plants have been addressed for their bioactive compounds [28]. As EOs are a coevolutionary product of plants, functioning to protect them from herbivore attack, they often elicit undesirable and potentially harmful effects on animal cells and bodily functions [5]. However, these undesirable effects of EOs can be exploited and used to treat diseases and symptoms. Examples include emetics and laxatives, muscle relaxants, cardiac stimulants, and cardiac depressants resulting in hypotension and induction of bradycardia [8].

Atherosclerosis is the arterial build-up of fats and other compounds and is a large contributor to thrombosis and arterial occlusion [29]. The main driver of this disease is the oxidation of low-density lipoproteins (LDLs), and it was shown that phenolic-rich EOs such as thymol and eugenol exhibit the highest LDL antioxidative effect, with their capabilities increased through also reducing LDLs' affinity for the LDL receptor [7]. Other benefits for treating cardiovascular disease, thus reducing the risk of atherosclerosis, include the reduction of cholesterol and triglyceride levels in plasma, in which black cumin oil achieved this reduction in rats over a period of 12 weeks, with low toxicity and no adverse effects in kidneys or liver [7]. Additionally, recent studies have demonstrated the capability of EOs to act on inflammatory and other cellular processes associated with cardiovascular diseases, by preventing the secretion of proinflammatory factors through the reduction of lipopolysaccharide (LPS) [30, 31]. EOs may be used in both analgesics and anti-inflammatories, such as black cumin and eucalyptus oils [32, 33]. It is clear, with respect to recent research, that Eos' ability to bind various cellular receptors has therapeutic value and potential for both treatment of infectious diseases, and for inborn and intrinsic diseases. Importantly, these mechanisms of action of EOs leading to cellular and metabolic responses make them attract new sources of anticancer therapeutic strategies.

The aim of this review is to evaluate the research behind the application of EOs as anticancer agents, both* in vitro* and* in vivo*. Cancer cell target specificity without noncancerous tissue toxicity will be explored, as well as the use of EOs in combination in conventional chemotherapeutic strategies.

## 2. Anticancer Proprieties of EOs

According to the International Agency for Research on Cancer (IARC), in 2012 there were 14.1 million new cancer cases worldwide and 8.2 million cancer deaths [34]. Cancer is now the leading cause of death and is expected to increase by 70% in the next two decades, with lung, liver, stomach, colorectal, breast, prostate, and oesophageal cancer accounting for most of the deaths [34, 35]. These statistics support the need for new and novel chemotherapeutic drugs in the coming years.

Cancer is broadly divided into three stages: (1) initiation, in which cellular DNA damage and mutation occur on carcinogen exposure and due to failure of DNA repair mechanisms; (2) promotion, in which hyperproliferation, tissue remodelling, and inflammation occur due to expansion of initiated cell/s; and (3) progression, in which preneoplastic cells form tumors through clonal expansion, further facilitated by an increase in genomic instability and altered gene expression [36]. The different stages of carcinogenesis require different chemotherapeutic approaches, due to the evolutionary nature of cancer, which lead to alterations in sensitivity to therapy. Specifically, tumour progression is associated with genomic instability, through accumulation of mutations for factors involved in cell proliferation, apoptosis, and DNA repair, amongst others [36, 37]. Chemotherapy drugs act on the promotion stage, in ways including cellular proliferation inhibition, increased rate of cell death, and induction of tumor cell differentiation [38].

Although research on the application of EOs as anticancer therapeutic agents is relatively new, approximately half of conventional chemotherapy agents have plant origin, with roughly 25% directly derived from plants, and 25% being chemically modified versions of phytoproducts [28]. One such molecule is paclitaxel. Paclitaxel (of which the most common brand name is Taxol) was originally derived from the bark of the tree* Taxus brevifolia* [39]. Its mechanism of action is based on the induction of a mitotic arrest via the targeting of the cytoskeleton component tubulin, resulting in mitotic checkpoint activation, and subsequent apoptosis [39]. It is used as a therapeutic agent either as a single agent or in combination therapy strategies for various cancer types, including ovarian, breast, and pancreatic cancer [39]. Laboratory synthesis of this drug was needed due to depletion of the natural source, primarily through a synthesis route involving EO constituent patchoulol (Figure 1) to produce patchoulol oxide [40]. More recently, Altshuler and collaborators found that the enantiomer (+)-citronellal, a major component of* Corymbia citriodora* and* Cymbopogon nardus* EOs, is also an effective microtubule-disrupting compound, similarly to better-known microtubule-disrupting agents colchicine and vinblastine [41].

**Figure 2:**
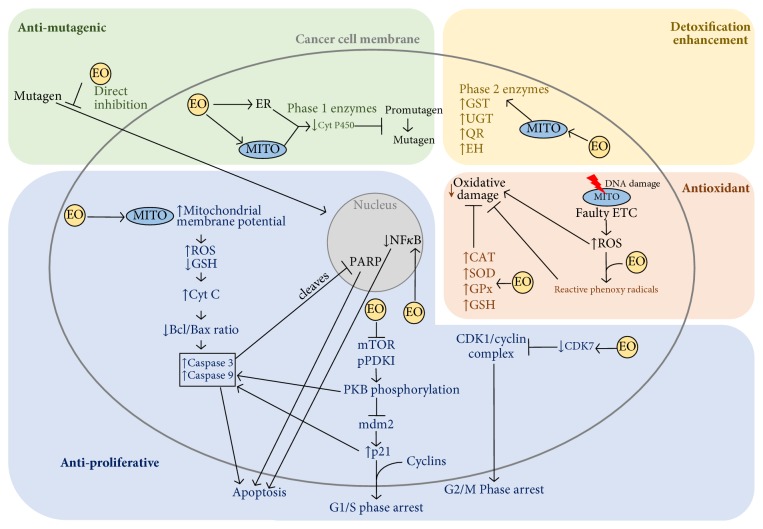
*Essential oils cancer preventative and anticancer mechanisms of action*. EOs possess antimutagenic, antiproliferative, antioxidant, and detoxifying capabilities acting on various pathways in the cancer cell as well as cancer preventative capabilities. EOs may directly inhibit mutagen entry into the cell. EOs can decrease phase I enzymes such as CytC, preventing mutagen formation, and increase phase II enzymes such as GST, UGT, QR, and EH for enhanced detoxification. EOs bind ROS forming reactive phenoxy radicals which bind further ROS and increase antioxidative enzymes CAT, SOD, GPx, and GSH thus preventing oxidative damage as a cancer preventative mechanism. EOs disrupt mitochondrial membrane potential causing an increase in ROS and decrease in GSH, release of CytC, resulting in a cascade of disruption in Bcl/Bax ratio, increase in caspase 3 and caspase 9 activity, and PARP cleavage, resulting in apoptosis. EOs suppress mTOR and pPDK1 causing PKB dephosphorylation, which dually acts to initiate caspase activity and deactivate mdm2, causing an increase in p21 to further initiate caspase activity resulting in apoptosis. Increased p21 also induces G1/S phase cell cycle arrest. EOs cause a decrease in CDK7, blocking CDK1/cyclin complex causing G2/M phase cell cycle arrest. Bax: B-cell lymphoma 2-associated X protein; Bcl-2: B-cell lymphoma 2; CAT: catalase; CDK: cyclin-dependant kinase; CytC: cytochrome C; CytP450: cytochrome P450; EH: epoxide hydrolase; EO: essential oil; ER: endoplasmic reticulum; ETC: electron transport chain; GPx: glutathione peroxidase; GSH: glutathione; GST: glutathione* S*-transferase; mdm2: murine double minute 2; mTOR: mechanistic target of rapamycin; MITO: mitochondria; NF*κ*B: nuclear factor-*κ*B; PARP: poly ADP ribose polymerase; pPDK1: protein pyruvate dehydrogenase kinase 1; PKB: protein kinase B; QT: quinone reductase; ROS: reactive oxygen species; SOD: superoxide dismutase; UGT: uridine 5′-diphospho-glucuronosyltransferase.

EOs have been shown to possess anticancer properties through various mechanisms, including cancer preventative mechanisms, as well as acting on the established tumor cell itself and interaction with the microenvironment (Figure 2) [7, 42].

### 2.1. Antimutagenic Proprieties and Detoxification Enhancement

EO cancer preventative mechanisms include direct inhibition of the mutagen entering the cell, although underlying mechanisms remain unexplained [7, 43]. Other cancer preventives and antimutagenic properties include a decrease of enzymes involved in drug metabolism. These include phase I enzymes such as cytochrome P450 [44, 45]. Phase II enzymes are responsible for detoxification and are mainly comprised of transferases [46]. Glutathione *S*-transferase (GST), uridine 5′-diphospho-glucuronosyltransferase (UGT), quinone reductase (QR), and epoxide hydrolase (EH) were observed to be increased on sulfur-containing EO activity such as that from garlic and onions [47–52]. The EO component citral, a monoterpene obtained from plants such as lemongrass, has been shown to induce phase II enzymes in a dose-dependent manner [53]. The mechanism of action of citral is due to its geranial isoform component [53]. Recent studies have shown citral to inhibit cell proliferation and tumor growth by increasing the intracellular levels of oxygen radicals and, consequently, inducing oxidative stress, leading to reduction of cancer cell proliferation and ultimately resulting in cell death [54, 55].

### 2.2. Antiproliferative Mechanisms of Action of EOs

Key hallmarks of cancer include resisting cell death, sustained proliferative signaling, and evading growth suppressors [28]. Therefore, therapeutic strategies focused on inducing apoptosis and cellular arrest are of clear significance. EOs have been shown to induce both the intrinsic (or mitochondria-dependent) and extrinsic (or death receptor-dependent) apoptosis pathways.

Girola and coworkers (2015) tested the antitumor properties of a camphene isolated from the EO of* Piper cernuum* in melanoma cells. The study demonstrated that this compound was able to induce apoptosis through the caspase-3 pathway activation, as well as activating the endoplasmic reticulum (ER) stress signaling [56]. Another study focused on the evaluation of the mechanism of action of carvacrol, a phenolic monoterpenoid abundant in the EOs of oregano and thyme [57]. In the metastatic breast cancer cell line MDA-MB-231, carvacrol induced apoptosis via mitochondrial membrane permeabilization, resulting in cytochrome C release, induction of caspases indicated through poly ADP ribose polymerase (PARP) cleavage, and DNA fragmentation [57]. Frankincense extracts obtained from* Boswellia sacra *induced PARP cleavage with apoptosis in MDA-MB-231 cells, with higher cancer cell specificity [14]. Citral was also shown to induce caspase activation and subsequent apoptosis induction in several cancer cell types, including colorectal cancer and glioblastoma [58–60]. Other studies have shown that citral treatment can lead to reduction of expression of prostemness and prosurvival factors such as aldehyde dehydrogenase 1A3 (ALDH1A3) and microtubule affinity regulating kinase 4 (MARK4) in cancer, respectively [61, 62].

PKB (Protein kinase B) is a key molecule with roles regarding cellular metabolism, transcription, cell cycle progression, and survival [63]. The vapor of* Litsea cubeba* seed oil induced cell cycle arrest and apoptosis of nonsmall cell lung carcinoma cells, a cancer type with a high mortality rate [64]. In this study, apoptosis occurred due to a significant decline in the expression of mTOR (mechanistic target of rapamycin) protein, and a decline in the phosphorylating ability of PPDK1 (protein pyruvate dehydrogenase kinase 1), leading to dephosphorylation of PKB and initiating the caspase-dependent apoptosis pathway [64]. Furthermore, PKB dephosphorylation inactivated mdm2 (murine double minute 2), leading to an increase in p21 expression, and subsequent caspase initiation after G1/S phase arrest [64]. This dual mechanism offers antiproliferative as well as antioxidant proprieties, and the vapor can be inhaled directly to the site of cancer in the lung, offering a clear advantage in administration [64].

Wu and colleagues showed that administering organosulphur components of garlic significantly decreased cell viability (*P* = <0.05) compared with control in a dose and time-dependent manner, with diallyl trisulphur being the most effective [65]. This was observed in J5 liver tumor cell line through a G2/M cycle arrest, leading to cell death* via* a decrease in expression of cyclin-dependent kinase (CDK) 7 and subsequent CDK1/cyclin complex inhibition [65].

Expression of NF*κ*B (nuclear factor-*κ*B) is abnormally increased in cancer cells and is particularly associated with cancer initiation and progression [66–68]. *α*-terpineol, a monoterpenoid alcohol, was able to downregulate the transcription of NF*κ*B in a range of tumor cells, with the strongest inhibitory effect on small cell lung carcinoma cell line NCI-H69 [69]. Finally, *α*-terpineol was further shown to have synergistic properties with another monoterpene, linalyl acetate, in colon cancer cells, inhibiting NF*κ*B expression and resulting in apoptosis [70].

### 2.3. Antioxidant Proprieties of EOs

Mitochondrial DNA damage can result from oxidative stress, and defects on the electron transport chain (ETC) result in the further release of reactive oxygen species (ROS) and further DNA, lipid, and protein damage [71]. Antioxidant properties of EOs can, therefore, contribute to cancer preventative mechanisms [36, 72]. Specific EO components such as eugenol, the main constituent extracted from clove oil, can react with ROS to form reactive phenoxy radicals, which can then combine with further ROS and prevent further damage [73]. Other cancer protective mechanisms induced by EOs include the induction of the expression of antioxidant enzymes such as catalase, superoxide dismutase, glutathione peroxidase, and glutathione, as shown by Manjamalai and Berlin Grace [74]. Treatment with EO extracts of* Wedelia chinensis* (96% of the components being carvacrol and trans-caryophyllene) lead to an increase in intracellular antioxidant activity, subsequently leading to a significant reduction in tumor mass volume as well and regeneration of surrounding healthy tissue [74].

However, research by Le Gal and colleagues (2015) showed that increased intracellular antioxidant activities can actually increase tumor cell survival, both using* in vitro* and* in vivo* models [75]. Specifically, oxidized glutathione, an indicator of oxidative stress levels, was increased on antioxidant administration, thus offering protection for the melanoma metastasis cancer cells [75]. This is a similar mechanism as the one observed with conventional chemotherapy drug methotrexate, which is a prooxidant and increases cellular glutathione levels [76]. Therefore, EO extracts with these types of antioxidant properties are likely to be more beneficial as chemopreventive agents for nontumor tissue.

Finally, Legault and colleagues (2000) showed that balsam fir oil extracts led to decreased glutathione levels, mediated by the EO component gamma-caryophyllene, which promotes ROS increase and glutathione decrease due to *α*-humulene in a dose-dependent manner [77].

## 3. Cancer Cell Specificity of Essential Oils

Conventional chemotherapy drugs are more cytotoxic to cancer cells due to their higher rate of cell division; however, due to this mechanism of action, there are issues with tumor cell specificity and associated cytotoxicity to healthy cells [78]. The subsequent side effects in the patient can hinder recovery and even prove to be life-threatening. Currently, combined therapeutic approaches of surgery followed by chemotherapy, radiotherapy, and immunotherapy offer increased chances of treating cancer and remission [78]. However, this does not address the need for cancer cell-specific therapy, or an increased therapeutic window between normal and cancer cells. Novel targeted strategies are a significant improvement but still have issues with cell specificity, and more importantly, a very high attrition when moving these agents from preclinical studies to clinical applications [78]. The use of monoclonal antibodies is highly selective, though it has limited cytotoxic activity [79]. Combined administration of monoclonal antibodies and conventional chemotherapy drugs is one potential route for solving this problem, delivering the highly cytotoxic agent specifically to cancer cells [79].

The use of EOs extracts as single agents has been shown in various* in vitro* studies to specifically target cancer cells, with absent or markedly less cytotoxicity exhibited towards healthy cells with a range of mechanisms of action (Table 1).

*Boswellia sacra *extracts have shown very promising results* in vitro* and* in vivo*.* Boswellia sacra* extracts were shown to be cytotoxic to three breast cancer cell lines (T47D, MCF7, and MDA-MB-231) at varying concentrations, which were noncytotoxic to immortalized normal human breast cells MCF10-2A [14]. This study also showed that* Boswellia sacra* extracts that were hydrodistilled for 12 hours at 100°C were more potent than the essential oil extracts prepared at 78°C, with a higher amount of boswellic acid present. Apoptosis markers activated caspase 3 activity, PARP cleavage, and DNA fragmentation rapidly in MDA-MB-231 but not MCF10-2A cells [14]. Importantly, treatment with the extracts blocked the growth of multicellular tumor spheroids from T47D, indicating the potential for efficacy in* in vivo* models [14]. Similarly,* Boswellia sacra* showed cell-specific cytotoxicity in a dose-dependent manner to bladder transitional cell carcinoma cell line J82, in contrast to no cytotoxicity observed in normal bladder cell line UROtsa [17]. Treatment of J82 cells rapidly led to cell shrinkage and detachment from the plate, whereas no changes were observed for UROtsa cells. This effect was associated with decreased expression of 47 genes after treatment with the EO extracts, whose functions include transcription factors, cell cycle regulation, and cell proliferation [17]. Finally,* Boswellia sacra *also showed cytotoxicity towards human pancreatic cells, both cultured and in a xenograft mouse model, exhibiting repression of cell cycle regulators and activation of the caspase pathway in* in vitro* cultures, and causing decreased tumor cell growth and tumor cell death* in vivo* [80]. Similarly, to the work by Suhail et al. (2011) [14], EO extract potency was increased with the increase of hydrodistillation temperature, associated with the extraction of higher levels of boswellic acids and sesquiterpenes, which is indicated to be positively correlated with cytotoxicity [80].

EO extracts from* Amomum tsaoko* exhibited cytotoxicity towards various human cancer cell lines, including liver cancer (HepG2 and Bel-7402), cervical cancer (HeLa), gastric adenocarcinoma (SGC-7901), and prostate cancer (PC-3) [15]. Importantly, these extracts were less effective towards normal hepatocytes HL-7702 and umbilical vein endothelial (HUVEC) cell lines [15]. The individual components of this EO mixture, eucalyptol and geraniol, were also tested [15]. Eucalyptol was not cytotoxic to any cancer cell line, and geraniol exhibited a minimal cytotoxic effect towards all cancer cell lines but was markedly lower than the complete EO mixture [15]. Synergism of eucalyptol and geraniol with each other and/or other EO components, therefore, must contribute to the cytotoxic activity [15].

## 4. Synergism of EO Extracts with Conventional Chemotherapeutic Agents: Potential of Combination Therapy Using EOs

Specific EO constituents have been shown to enhance the cytotoxic activity of chemotherapy drugs in various cell lines (Table 2), thus increasing the therapeutic window, that is, lowering the required drug concentrations whilst providing the same effect [22, 23].

Docetaxel is the first line therapy for hormone-refractory prostate cancer, which has a median survival of 20 months [22]. Docetaxel is associated with serious side effects and is currently used in combination with treatment exhibiting dose-dependent toxicity to the patient [22]. *d*-limonene showed cytotoxic activity alone towards prostate cancer cell line DU-145, and when administered alongside docetaxel, sensitized the cells towards this drug in a dose-dependent manner allowing for a markedly lower dose of docetaxel to be used, achieving the IC_50_ in concentrations from 2.8 nM to 1.9 mM [22]. Limited toxicity was also shown towards normal prostate epithelial cells. Further analysis on the effects of combined treatment showed an increase in ROS production from both mitochondrial dependent and independent pathways, as well as increased cytochrome C release, p53 stabilisation, and caspase and PARP cleavage after 0-48 hours [22]. In addition to decreasing the amount of toxic docetaxel required,* d*-limonene showed low toxicity towards humans. It is possible that this combination may also be effective in docetaxel-resistant cell lines [22].

*β*-caryophyllene, which was not cytotoxic as a single agent, was shown to markedly increase the cytotoxic activity of paclitaxel in various cancer cell lines (Table 2). Specifically, the largest effect was observed on DLD-1 cells treated with paclitaxel combined with 10 *μ*g/mL^−1^ *β*-caryophyllene, increasing paclitaxel activity approximately 10 times [23]. It was shown that *β*-caryophyllene increased cell membrane permeability for paclitaxel uptake, likely due to *β*-caryophyllene accumulation in the lipid bilayer, and thus altering the permeability for substances such as paclitaxel [23].

Neutropenia is a common side effect of both cancer itself and therapies including chemotherapy and radiotherapy, the latter especially if targeted to active sites of bone marrow proliferation [81]. Cancer-related neutropenia has a high mortality rate due to susceptibility to infectious diseases, particularly from gram-negative bacterial infections, and combined with fever is considered an oncological emergency [81]. Currently, there are limited adjunctive treatments, one of which is the administration of granulocyte colony-stimulating factors (G-CSFs), in selected patients only, which promotes bone marrow production of granulocytes [81]. Alternatively, chemotherapy dose-modification may be deemed appropriate [81]. A study by Zhuang and coworkers (2009) which included 105 cancer patients with nonterminal breast, colorectal, nasopharyngeal, or lung cancer showed significant results in preventing the depletion of leukocytes (14.2%) and neutrophils (11%), versus control (29.1%) over a 6-week period [82]. Flow cytometry analysis showed a larger depletion of CD4 and natural killer cells in the placebo receiving group versus the Chinese medicinal herb complex (CCMH) receiving group [82]. The largest component of the CCMH was the EO component citronellol (273.6 mg per capsule), a known strong antioxidative compound, also exhibiting anticancer and anti-inflammatory properties, as well as promoting wound healing [82]. It is not clear from this study how exactly citronellol and each other component contributed to results. So, to date the mechanism of action remains to be elucidated.

Geraniol has been shown previously to sensitize cancer cells to the conventional chemotherapeutic agent 5-fluorouracil (5-FU), also causing an increased uptake of the drug [83, 84]. Geraniol has also been shown to be chemoprotective towards normal colon cells in rats when administered with the potent carcinogen dimethylhydrazine [85]. This effect occurs through mediating the reduction of DNA damage when compared with controls where no EO extract was used [85].

## 5. Conclusions and Future Directions

EO have been shown to possess a wide range of anticancer properties and mechanisms. Considering the myriad of components present and the mechanism and synergistic capabilities of EO extracts, it is of paramount importance to perform further studies regarding evaluation on how EO minor components contribute to the overall effect of the EO extract mixture. Further* in vitro* and* in vivo* research into achieving the most effective cytotoxic EO mixture composition would allow for more targeted therapy, and with increased specificity to cancer cells over non-cancer tissue. Furthermore, the currently used concentrations of conventional chemotherapy drugs could potentially be reduced combined with specific EO, which could also decrease chemotherapy-associated toxicity. Moreover, synthetic modification of these molecules may allow improving their overall efficacy further. However, there is still a significant lack of preclinical studies for EOs as anticancer agents; thus many EOs require further safety and toxicity studies before they can take part in clinical trials.

Cancer cell specificity is a sought-after propriety that is lacking in conventional chemotherapeutic strategies [79, 86]. As well as addressing cellular specificity, another strategy to increase cell specificity includes novel drug delivery strategies [86]. Specifically, a new field addressing this involves the use of microspheres made of proteins or synthetic polymers containing the anticancer agent or EO, for delivery to the specific organ or another site of cancer [86]. These can be administered intravenously or intra-arterially depending on the target site [86]. The use of microspheres has promising potential due to multiple types of drugs being successfully contained and delivered in a single vehicle, offering the potential for combination therapies, but also, the use of nanoemulsions is an improvement to transport and to deliver the EOs with anticancer properties, improving their therapeutic effect [87]. Cancer cell specificity can also be enhanced by the use of ligands added to the surface, targeting overexpressed cell surface proteins on the cancer cell [88]. Crucially, EOs can be degraded through physical, chemical, or enzymatic processes, so microsphere encapsulation may prevent this for optimised delivery [88, 89]. This way, EOs and other drugs may be released in a controlled manner, potentially reducing excess dosage and increasing the overall safety of these constituents, and offering a promising strategy for targeted drug and EO delivery to cancer cells [89].

In conclusion, although this is a relatively new and emerging area of cancer research, the ability of EOs and their components of having such diverse anticancer effect through acting on various pathways and cellular mechanisms is compelling. Thus, it is warranted that more studies be performed to expand the present knowledge of these mechanisms with the aim of promoting cell-specific and individualized cancer therapy.

## Figures and Tables

**Table 1:** Essential oils bearing plants and main constituents with targeted cancer cell cytotoxicity in *in vitro* studies.

Species	Major EO constituent(s)	Cancer cell lines	Noncancer cell lines	Major findings and EO concentrations	Mechanisms	REF
*Thymus fallax*	Carvacrol, p-cymene, thymol and *γ*-terpinene	DLD-1 (CRc)	Mouse fibroblast (L.929)	Cytotoxic to cancer cells (IC_50_ 0.347 mg/mL) and noncytotoxic to normal cells (IC_50_ 22 mg/mL)	Antioxidant activity	[13]

*Boswellia sacra*	*α*-pinene, *α*-thujene, *β*-pinene, myrcene and boswellic acid	T47D, MCF7, MDA-MB-231 (Bc)	Immortalized normal human breast (MCF10-2A)	Cytotoxic to cancer cells (EO dilution IC_50_ 1 : 900 for TD47, 1 : 1000 for MCF7, 1 : 950 for MDA-MB-231) and noncytotoxic to immortalised normal cells (EO dilution IC_50_ 1 : 680)	Antiproliferative	[14]

*Amomum tsaoko*	1,8-cineole, *ρ*-propylbenzaldehyde, geraniol, geranial, *α*-terpineol, *α*-phellandrene, neral and *β*-pinene	HepG2 and Bel-7402 (Lc) HeLa (Cc), A549 (Lc), SGC-7901 (GAC), PC-3 (Pc)	Hepatocyte (HL-7702) and umbilical vein endothelial (HUVEC)	Cytotoxic to cancer cells, particularly HepG2 (IC_50_ 31.8 *μ*g/mL), Hela (IC_50_ 66.46 *μ*g/mL) and Bel-7402 (IC_50_ 96.08 *μ*g/mL), with less cytotoxicity towards HL-7702 (IC_50_ 272.4 *μ*g/mL) and HUVEC (IC_50_ 163.91 *μ*g/mL). No cytotoxicity towards A549	Antiproliferative	[15]

*Lippia alba *(Citral chemotype)	Geranial, neral, geraniol, *trans*-*β*-caryophyllene, 6-methyl-5-hepten-2-one, limonene, linalool	HeLa (Cc)	African green monkey kidney (Vero)	Cytotoxic to cancer cells (CC_50_ 3.5 *μ*g/mL) and noncytotoxic to normal cells (CC_50_ > 100 *μ*g/mL)	Citral-dependent cytotoxicity	[16]

*Boswellia sp. *(1,200 mg/ml frankincense gum resin)	Duva-3,9,13-trien-1,5alpha-diol-1-acetate, octyl acetate, o-methyl anisole, naphthalene decahydro-1,1,4a-trimethyl-6-methylene-5-(3-methyl-2-pentenyl), thunbergol (Mikhaeil et al., 2003)	J82 (Blc)	Human urothelium (UROtsa)	Cytotoxic to cancer cells (no viable cells after EO dilution 1 : 1,100 after 24 hours) and noncytotoxic to normal cells (no viable cells after EO dilution 1 : 400)	Antiproliferative	[17]

*Casearia sylvestris*	Bicyclogermacrene, *β*-caryophyllene, spathulenol, *α*-humulene, *α*-pinene	HeLa (Cc), A549 (Lc) HT-29 (CRc)	Monkey kidney (Vero) and mice macrophages	Cytotoxic to HeLa (CD_50_ 63.3 *µ*g·ml^−1^), A549 (CD_50_ 60.7 *µ*g·ml^−1^) and HT-29 (CD_50_ 90.6 *µ*g·ml^−1^) with less cytotoxicity to Vero (CD_50_ 210.1 *µ*g·ml^−1^) and macrophages (CD_50_ 234.0 *µ*g·ml^−1^)	Cytotoxicity	[18]

*Zanthoxylum rhoifolum* Lam	ß-caryophyllene, *α*-humulene, *α*-pinene, myrcene and linalool	HeLa (Cc), A549 (Lc) HT-29 (CRc)	Monkey kidney (Vero) and mice macrophages	Cytotoxic to HeLa (CD_50_ 90.7 *µ*g/ml), A549 (CD_50_ 82.3 *µ*g/ml), and HT-29 (CD_50_ 113.6 *µ*g/ml) and noncytotoxic to normal cells (CD_50_ > 600 *µ*g/ml)	Cytotoxicity	[19]

*Commiphora gileadensis*	Sabinene, ß-caryophyllene, germacrene D, *α*-pinene	BS-241 (Mouse T-cell lymphoma) MoFir (Epstein Barr virus transformed human B lymphocytes)	Normal human skin fibroblasts (FB)	EO dilution of 1 : 5000 killed 87% of BS-24-1 cells and 40% of MoFir cells	Antiproliferative	[20]

*Aniba rosaeodora*	Rosewood essential oil (REO), linalool	A431 (Ec), HaCaT (pre-cancerous)	Epidermal keratinocytes (HEK001, NHEK)	Cytotoxicity to cancer cells A431 and HaCaT (<20% viability) and minor cytotoxicity to normal cells HEK001 and NHEK (>70% viability)	Cytotoxicity	[21]

*Note*. Cytotoxicity is expressed as the concentration of the essential oils inhibiting cell growth by 50%; CRc: colorectal cancer; Bc: breast cancer; Lc: lung cancer; Cc: Cervical cancer; GAC: gastric adenocarcinoma; Pc: prostate cancer; BLc: bladder carcinoma; Ec: epidermoid carcinoma; IC_50_: inhibitor concentration 50; CC_50_: cytotoxic concentration.

**Table 2:** *In vitro* studies of essential oils in combination with conventional chemotherapy agents.

Cell lines	Chemotherapy drug used alone and concentration	EO constituent used alone and concentration	Combined EO and chemotherapy drug		Reference
Prostate cancer cell (DU-145)	Docetaxel IC_50_ 2.8 nM	*d*-limonene IC_50_ 2.8 mM	IC_50_ docetaxel 1.9 mM and d-limonene 0.2 mM		[22]

Human breast cancer (MCF-7)	Paclitaxel 0.025 *µ*g/mL^−1^ resulted in 28% cell growth inhibition	*β*-caryophyllene resulted in no inhibition of cell growth	*β*-caryophyllene 2.5 *µ*g/mL^−1^ and Paclitaxel 0.025 *µ*g/mL^−1^ resulted in 50% cell growth inhibition	*β*-caryophyllene 10 *µ*g/mL^−1^ and Paclitaxel 0.025 *µ*g/mL^−1^ resulted in 68% cell growth inhibition	[23]

Human colorectal adenocarcinoma (DLD-1)	Paclitaxel 0.025 *µ*g/mL^−1^ resulted in 17.3% cell growth inhibition	*β*-caryophyllene resulted in no inhibition of cell growth	*β*-caryophyllene 2.5 *µ*g/mL^−1^ and Paclitaxel 0.025 *µ*g/mL^−1^ resulted in 91% cell growth inhibition	*β*-caryophyllene 10 *µ*g/mL^−1^ and Paclitaxel 0.025 *µ*g/mL^−1^ resulted in 189% cell growth inhibition	[23]

Mouse fibroblast (L-929)	Paclitaxel 0.025 *µ*g/mL^−1^ resulted in 18.4% cell growth inhibition	*β*-caryophyllene resulted in no inhibition of cell growth	*β*-caryophyllene 2.5 *µ*g/mL^−1^ and Paclitaxel 0.025 *µ*g/mL^−1^ resulted in 36% cell growth inhibition	*β*-caryophyllene 10 *µ*g/mL^−1^ and Paclitaxel 0.025 *µ*g/mL^−1^ resulted in 123% cell growth inhibition	[23]
